# Optical Biosensors and Their Applications for the Detection of Water Pollutants

**DOI:** 10.3390/bios13030370

**Published:** 2023-03-10

**Authors:** Marcela Herrera-Domínguez, Gesuri Morales-Luna, Jürgen Mahlknecht, Quan Cheng, Iris Aguilar-Hernández, Nancy Ornelas-Soto

**Affiliations:** 1 Tecnológico de Monterrey, Escuela de Ingeniería y Ciencias, Ave. Eugenio Garza Sada 2501, Monterrey 64849, Mexico; 2 Departamento de Física y Matemáticas, Universidad Iberoamericana, Prolongación Paseo de la Reforma 880, Mexico City 01219, Mexico; 3 Department of Chemistry, University of California, Riverside, CA 92521, USA

**Keywords:** optical biosensors, water pollutants, water monitoring, interferometers, resonators, SPR biosensor, fiber optic biosensors, emerging contaminants, heavy metals in water, waterborne pathogens

## Abstract

The correct detection and quantification of pollutants in water is key to regulating their presence in the environment. Biosensors offer several advantages, such as minimal sample preparation, short measurement times, high specificity and sensibility and low detection limits. The purpose of this review is to explore the different types of optical biosensors, focusing on their biological elements and their principle of operation, as well as recent applications in the detection of pollutants in water. According to our literature review, 33% of the publications used fluorescence-based biosensors, followed by surface plasmon resonance (SPR) with 28%. So far, SPR biosensors have achieved the best results in terms of detection limits. Although less common (22%), interferometers and resonators (4%) are also highly promising due to the low detection limits that can be reached using these techniques. In terms of biological recognition elements, 43% of the published works focused on antibodies due to their high affinity and stability, although they could be replaced with molecularly imprinted polymers. This review offers a unique compilation of the most recent work in the specific area of optical biosensing for water monitoring, focusing on both the biological element and the transducer used, as well as the type of target contaminant. Recent technological advances are discussed.

## 1. Introduction

Detecting pollutants in water bodies accurately is crucial for quantifying their impact and developing tailored strategies to reduce their effects. Due to the variable complexity of environmental water samples, as well as the low concentrations at which some pollutants are found, so far chromatographic techniques are the gold standard for analytical detection [1,2]. However, biosensors have positioned themselves as a good alternative to these classical techniques. According to Markets and Markets, in 2021 the biosensors market was valued at USD 25.5 billion and is projected to reach USD 36.7 billion by 2026 [3].

Currently, biosensors primarily serve in the medical fields and life sciences, but their use has been extended to the food industry [4], biotechnology [5] and environmental monitoring [6], the latter which will be discussed in depth in this review. Biosensors are analytical devices that use biological recognition elements connected to transducers to generate a signal in response to a specific reaction between two elements [7]. This reaction is proportional to the concentration of chemical components present in the sample. There are numerous approaches to develop a biosensor, but overall, a biosensor can be classified as electrochemical, piezoelectric, optical, mechanical, and thermal, depending on the type of transducer used.

Optical biosensors stand out because they can provide valuable information about a sample (e.g., kinetic behavior, concentration, molecular interaction, etc.) while avoiding electrical or magnetic interference [8]. Moreover, optical biosensors are highly sensitive and can detect analytes even at attomolar [9] and femtomolar [10] concentrations. Despite the aforementioned advantages, electrochemical biosensors are still the most commercialized type of portable biosensor, mostly because they are easier to miniaturize.

The main challenges for the development of portable commercial optical biosensors are (a) device miniaturization, (b) stability of the biological recognition element, and (c) device reusability. Nevertheless, there are successful benchtop commercial optical biosensors used for drug discovery, small molecule, and therapeutic screening, among which the Biacore system and its iterations have been in production since around 2004 [11]. These biosensors are not commonly for environmental applications, although research advances could bring breakthroughs in this area.

Recently, optical sensors and biosensors have been developed that take advantage of the optical elements of smartphones to capture signals and transform them into measurable values. For example, high-resolution cameras allow for data acquisition, while exposure lights provide the sources of light excitation [12]. Hence, the optical characteristics of an image, such as color, luminescence, pixel counts, reflected light, and scattered light can be processed to obtain relevant information [13]. Smartphone-based optical sensors and biosensors use colorimetric [14], fluorescence [15] or bright-field imaging [16] as their detection principle; due to the complexity of this data, tools such as machine learning and deep learning are used for processing [12]. Within the biosensors that have been developed with this technology, most of them have been used for disease diagnosis [17] and point-of-care analysis [15]. However, their working principles can also be extended to environmental monitoring. For example, a smartphone has been used to detect the fluorescence emitted by labeled antibodies to quantify bisphenol A in lake and tap water [18]. Although further research is still required for the development of portable and accessible biosensors using this technology, the progress achieved so far represents a breakthrough in the miniaturization of optical devices and their potential use for on-site water monitoring.

The aim of this literature review is to analyze the most recent works published on the specific topic of optical biosensors for the detection of pollutants in water, since information is needed on fast, simple and in situ methodologies that can be used even by users without highly specialized training to increase their awareness of the type and concentration of pollutants found in water bodies and incentivize regulatory measures.

At present, review articles have been published on optical biosensors, but these are mostly focused on biomedical sensing applications such as biomarkers, disease detection [17] and point-of-care analysis [13]. Similarly, although to a lesser extent, biosensors have been used in the food industry [2].

Unlike the few reviews that broadly focus on biosensing water pollutants [19,20,21], this paper purposely excludes other types of biosensors such as electrochemical, mechanical, piezoelectric, etc., and instead seeks to expand the information pertaining exclusively to optical biosensors published in the last decade. Optical biosensors are highly promising for environmental analysis because of their versatility in terms of configuration and types of target samples and the fact that optical devices stand out from other types of transducers because of their immunity to external signals [22].

This review discusses recent advances in the development and application of optical biosensors for the environmental monitoring of water samples (freshwater and wastewater), focusing on the detection of pesticides, pharmaceuticals, microorganisms, toxins and heavy metals. A brief description of the main types of biological detection elements is given, as well as a discussion of their advantages and limitations. Subsequently, the different types of optical transducers currently available and their working principle are described, including some of the progress made in their different configurations. Next, papers from the last decade on the subject of optical biosensors for water monitoring are presented. Works are detailed, with an emphasis on each of the elements that conform the biosensor (i.e., transducer and biological element), specific type of sample (e.g., freshwater, wastewater) and analytical parameters.

## 2. Methods

The literature review was conducted using the following databases: Pubmed, ScienceDirect and Scopus. The search was delimited to last decade and the following search terms were used: “optical biosensors”, “environmental monitoring”, “water samples”, “resonators”, “interferometer”, “grating biosensors”, “SPR”, “refractometers”, “fiber optic biosensors”, “immunoassay”, “pharmaceuticals in water”, “emerging contaminants”, “heavy metals”, “waterborne pathogens”, “DNA biosensors”, and “whole cell biosensors”. A total of 46 original research articles were considered for analysis. Once the articles were collected, they were classified according to the type of contaminant detected. For each article the type of transducer used, the biological element, the analyte of interest and the type of water sample in which it was tested was identified.

## 3. Main Optical Biosensor Components

### 3.1. Biological Recognition Element

#### 3.1.1. Enzymes

Enzymes are among the most used biomolecules in the development of biosensors. A main advantaged offered by enzymes is that their production can be easily scaled up [23]. Nonetheless, enzymatic activity can be affected by temperature and pH changes, and they are susceptible to denaturization. These challenges can be solved by immobilizing enzymes on substrates or trapping them in polymer networks [23]. An important part of the research and development of enzyme biosensors is focused on long-term stability and reproducibility studies [24].

#### 3.1.2. Antibodies

Antibodies are a type of protein produced by the immune system to recognize and attack infections. In a biosensor, the chemical interaction between an antigen and an antibody produces a signal proportional to their concentration in the sample, which is then captured by a transducer [25]. Antibodies can be produced for any type of target molecule, including molecules with low molecular weights [26]. However, the affinity and specificity of an antibody can be affected during immobilization, impacting the overall biosensor performance [27].

#### 3.1.3. DNA

Biosensors with DNA or RNA as the biological recognition elements are called genosensors. Genosensors can have high selectivity to interact with their complementary polymerase chains, and the sensitivity of the biosensor is improved by increasing the chain length of the genetic material [28]. These biosensors can be used to detect fractions of DNA/RNA or other types of biomolecules or chemical compounds [29].

Nucleic chains can be prepared using polymerase chain reaction (PCR), which is simple and does not involve the use of hazardous material [30]. Genosensors are limited by the number of molecules with which the genetic material can interact. In addition, hybridization processes can take days, hampering their use in commercial and real-time monitoring settings [30]. Therefore, most genosensors are developed for clinical applications.

#### 3.1.4. Other

Other biological elements used in the development of biosensors include tissues, whole cells, non-enzymatic proteins, and fatty acids.

As the name implies, whole-cell biosensors use the entire cell as a recognition element rather than isolated or purified components (e.g., enzymes, nucleic acids). Using the whole cell as a recognition element can decrease biosensor specificity and slows down the response time [31]. Still, they can interact with various types of target compounds and are versatile when integrated into different device configurations [32]. For example, the non-enzymatic proteins ERα and ERβ estrogen have been used not only for the detection of their target hormones [33], but also to target endocrine disrupting compounds in water [34]. Other examples are the use of lectins from Concanavalin A for pathogen detection [35], and stearic acid as a recognition element for copper [36].

In recent decades, whole-cell bioreporters (WCBs) have emerged as a low cost, high specificity, high sensitivity, and rapid alternative to biological receptors. These are living microorganisms with chromosomes or plasmids that have a regulatory promoter and a promoterless reporter either naturally present in the cells or added by genetic modification [37]. The protein encoding the reporter gene produces detectable signals when the regulatory promoter is active or repressed by a particular chemical or environmental stress [37]. WCBs are divided into class I, II and III based on which parameters are detected and how they are transformed into a measurable signal: those that react specifically to a type of target compound by increasing the output signal (class I), those that react specifically to stress conditions by increasing the output signal (class II), and those that react specifically to compounds or stress by decreasing the output signal (class III) [38]. WBCs have been shown to have excellent suitability for use in biosensors for water monitoring. Class I are the most widely employed for the detection of contaminants in water, although Class II and III have also been used to a lesser extent [39].

Molecularly imprinted polymers (MIP) are synthetic receptors in which specific recognition sites are formed via synthesis with a target template [40]. The resulting MIP is therefore capable of selectively recognizing the target analyte in the template-derived sites. MIPs have been developed to overcome the stability limitations affecting enzymes, as well as the high cost of producing antibodies.

#### 3.1.5. Comparison of Biological Elements for Optical Sensors Monitoring Water Quality

Table 1 compares the main biological recognition elements in terms of their affinity for an analyte, specificity, sensibility, stability, versatility, and cost.

Enzymes were classified as medium to high specificity (Table 1) as some enzymes can react with molecules that have similar structures to their substrate, so their use can be expanded to more than one target molecule. In water monitoring, this medium/high specificity can be exploited for the simultaneous detection of more than one pollutant. For example, the enzymatic detection of pesticides has been performed via acetylcholinesterase [41,42], halogenated compounds have been detected using haloalkane dehalogenases [43,44] and acid phosphatase has been used for heavy metals [45]. Despite this, according to our literature review, enzymes represent only 12% of the biological material used in the development of optical biosensors for water quality.

According to the data collected in this review, 42% of biosensors use antibodies as their recognition element. Antibodies are the preferred recognition element used for the detection of emerging pollutants such as pesticides [46], pharmaceuticals [47], and miscellaneous organic compounds [48,49]. They have also been used for monitoring pathogenic microorganisms [50] and toxins [51].

In the case of genetic material, as is well known, nucleic bases bind only adenine-thymine and cytokine-guanine. The combinations of these base pairs make DNA the receptor with the highest affinity and selectivity. However, this also limits their versatility and their potential applications in water quality. So far, DNA-based biosensors have been employed for the detection of pathogenic microorganisms and heavy metals in water, the latter of which is due to their mutagenic capacity on DNA. Genosensors have also been developed for detecting organic compounds such as bisphenol A [52], and Kim et al. developed a biosensor combining an aptamer with antibodies for the detection of tetracycline [53].

As for cells, being a complete system allows them to interact with more than one molecule, which detracts from their specificity but adds versatility. Cells have been shown to be able to detect different types of contaminants in water. So far, yeasts cells, microalgae and bacteria have contributed to the detection of pharmaceuticals [54], pesticides [55], and heavy metals in water [56].

MIPs can have high affinity and specificity and have the advantage of being more stable and having a low cost compared to natural receptors [57]. Their main uses in water quality biosensors are in the detection of emerging pollutants, mainly in pharmaceuticals, and MIPs have been shown to have detection limits close to those of antibodies [47,58]. Research and development of MIPs is emerging, but they could be a viable alternative for the mass production and commercialization of biosensors in the future.

In Table 1, sensitivity was considered as a function of the limit of detection (LOD). LOD ranges in terms of ng L^−1^ were obtained from the articles discussed in this review. It is important to note that for each biological element, these limits are variable and depend on the type of transducer employed in the biosensor. For example, in the case of enzymes, the reached upper limit (Table 1, 12,000 ng L^−1^) corresponds to a fluorescence-based biosensor, while the lower limit is for a resonator (Table 1, 1 × 10^−4^ ng L^−1^). This shows that both the biological element and the transducer have a direct impact on the final performance of the biosensor, so choosing the right components is critical.

### 3.2. Optical Transducer

#### 3.2.1. Interferometer

Interferometry is based on the superposition of a pair of light beams with different optical paths (space or dielectric media acting as waveguides) to generate controlled interferences. Interferometry is able to determine changes in the thickness or refractive index of a surface; any change in the refractive index of the bulk or adsorption of a biocoating induces changes in the intensity or phase of the resulting signal [59]. The light beams must be coherent with each other; in other words, they must come from the same source, be monochromatic and have the same frequency. Because of the interference produced, there is a change in the intensity of the resulting light, which depends on the optical path of the beams [60]. The first configurations used in the development of interferometers were by Mach-Zehnder and Young. Other configurations for interferometers are the Fabry-Perot Interferometer (FPI), the Exposed-Core microstructured optical fiber (ECF) and Reflectometric Interference Spectroscopy (RIfS).

**Figure 1:**
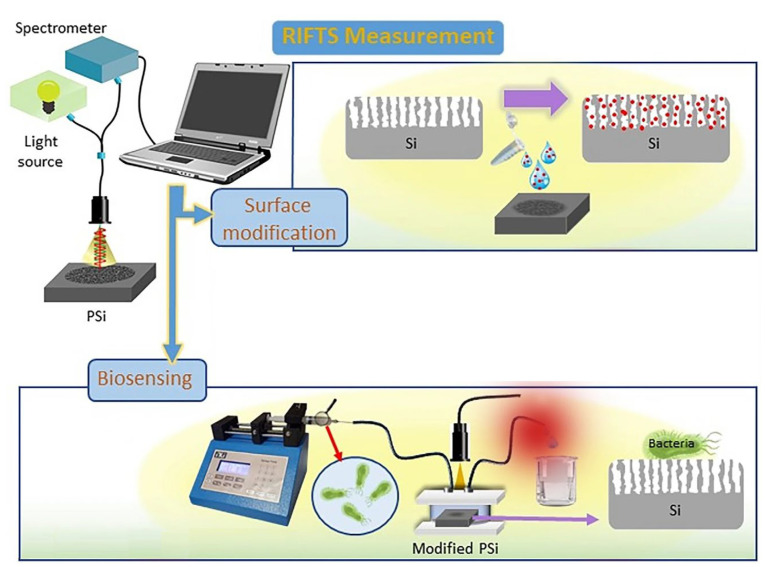
Schematic representation of a reflective interferometric Fourier transform spectroscopy (RIFTS) set up. During surface modification, analyte molecules enter the Si pores. Biosensing monitoring of a bacterial suspension is carried out in a fluidic system. Reproduced from [35].

Different interferometers have been designed for water monitoring. For example, Yaghoubi et al. used an interferometer based on RIfS (Figure 1), whose performance was improved using the Fourier transform (RIFTS). The work describes how they use porous silicon (PSi) substrates functionalized with lectins for the detection of *S. aureus* and *E. coli*, two pathogenic microorganisms commonly found in drinking water [35].

#### 3.2.2. SPR and LSPR

Surface Plasmon Resonance (SPR) is a phenomenon which occurs when the free electrons of a metal are excited by photons, creating an evanescent wave. This is useful for monitoring changes in the refractive index since the evanescent wave is highly sensitive to changes in the vicinity of the surface [61]. SPR occurs only at the nanometer scale in metals (gold and silver are preferred) and are divided into two types: Localized Surface Plasmon Resonance (LSPR) when the phenomenon occurs in metallic nanoparticles and SPR when it occurs in a metallic film [62]. The plasmon generated depends directly on the size of the nanoparticle or metal film and the material used. As for the material, it is known that from the physical point of view, silver is better since its plasmon is more intense. However, gold is more used because it is chemically inert [63]. One way to take advantage of the characteristics of each material is the use of multilayers or core-shell nanoparticles. It has been shown that metallic multilayer sensors have a wider measuring range and also an improvement in sensitivity compared to single-layer sensors [64]. Examples of the use of more than one metal in SPR substrates are the Ti/Ag/Au combination [64] and the triple layer composed of Au/Ag/Au [65]. In terms of size, the intensity of the plasmon varies according to the thickness of the metal layer or the diameter of the nanoparticles. For SPR propagation, it has been shown that a thickness of 40 nm generates the highest plasmon intensity, while for nanoparticles, the smaller the diameter, the lower the intensity [66].

The simplest SPR/LSPR biosensor includes only a gold layer [67,68]. Nevertheless, recently, the use of multilayers has been implemented, and such is the case of graphene, as this material has gathered interest for the development of SPR biosensors due to its optical and electrical properties. Incorporation of graphene to the SPR biosensors can be achieved via simple methodologies such as the addition of a layer of this material on the gold [69] or more complex arrangements such as the MoS2/Al/MoS2-graphene hybrid structure proposed by L. Wu et al. [70].

One of the benefits of LSPR is the wide variety of nanoparticle geometries available. Optical properties of nanoparticles depend on their size and shape; thus, tailoring these properties can improve the performance of the biosensor. A nanoparticle’s SPR can be modified throughout visible and near-infrared (NIR) wavelengths by changing its size, shape, or aspect ratio [71]. For example, spherical and rod nanoparticles have different optical properties and generate varying signal intensities; the surface plasmon shifts from the visible to the NIR region when the shape of nanoparticles is changed from spherical to rod [72]. In addition, nanoparticles with sharp tips such as triangles, stars, or pyramids show higher sensitivity towards refractive index changes and larger near-field enhancements [71]. Gold nanostars with branches and projecting tips have the plasmon in the NIR wavelength. In this case, LSPR signal intensity is proportional to the size of the tips, which improves the local electromagnetic fields significantly and results from the hybridization of the central core and tips plasmon resonance [73]. The most common nanoparticles are spherical, although there are also triangular, cubes, nanorods and nanostars. For use in SPR sensors, gold is preferred due to its biocompatibility, although silver or copper are also employed.

One of the most recent applications of plasmons is the use of surface plasmon coupled emission (SPCE), which improves the emitted fluorescence signal. Its operation is based on the near-field interaction of the fluorophore and the surface plasmon of the metal. It has remarkable optical qualities, such as high directivity, distinct polarization, wavelength resolution and background suppression. SPCE-based fluorescence is highly sensitive in the context of sensing and imaging [74], and has been successfully employed in optical sensors for detecting tannic acid in water, reaching detection limits at the picomolar scale [75]. Therefore, this is a promising technology that could improve the sensitivity of the environmental biosensors discussed herein.

#### 3.2.3. Optical Resonators

Optical resonators are a type of device in which photons are confined to a certain space. Once confined, the photons accumulate intensity due to interference, which in turn amplifies the signal [76]. When confined, light interacts with itself in a cavity, and only certain optical frequencies can be sustained without incurring significant losses. These are the so-called resonance frequencies. The microcavity functions as a transducer of optical signals and changes in the cavity alter the resonance parameters, which are converted into a change in light intensity [77]. In optical resonators, the sample interacts with light multiple times, which improves detection limits [76]. The Q factor is widely used in the evaluation of a resonator. This parameter describes the behavior of the resonator and is directly related to its geometry and material. The Q-factor has a critical role in determining the magnitude of the resonance shift and the resulting biosensing capacity. A larger cavity has a higher Q-factor. The Q factor of a smaller cavity is lower, but the resonance shift is higher [77].

This type of biosensors is developed in two configurations: Fabry–Perot resonators and ring resonators. The latter are the most common and consist of a circular structure (e.g., a micro-sphere, micro-disk, or micro-toroid), where the light is confined. Resonator biosensors can be found in arrangements such as silicon-on-isolator (SOI) [78], opto-fluidic ring resonator (OFRR) [79], subwavelength grating (SWG) [80] and whispering gallery mode (WGM) [81].

SOI are devices in which the ring is made of silicon. Due to the high refractive index of silicon, the optical modal field is strongly located near the surface of the waveguide, resulting in a high response to surface disturbances [78]. OFRR consists of a microtube that is functionalized on the inside, while a light source is outside. The main advantage of OFRR is that multiple analytes can be detected simultaneously; in addition, the amount of sample to be used is small and the results are very accurate [79]. SWG consists of silicon columns that are in the direction of propagation with a sub wavelength. One of the advantages of SWG is that the effective detection area is increased because in addition to the surface of the waveguide, the space between the silicon columns is also available. SWG offers a higher sensitivity and increased detection surface area and improves the overlap of biomolecules on the surface of the waveguide; that is, the sensitivity on the surface is high despite the accumulation of biomolecules [80]. WGM are found in the cavity as a result of total reflection at the exterior cavity contact. It has a low internal loss and, hence, a weakly constrained near-field, yet a greatly elevated Q factor [81].

**Figure 2:**
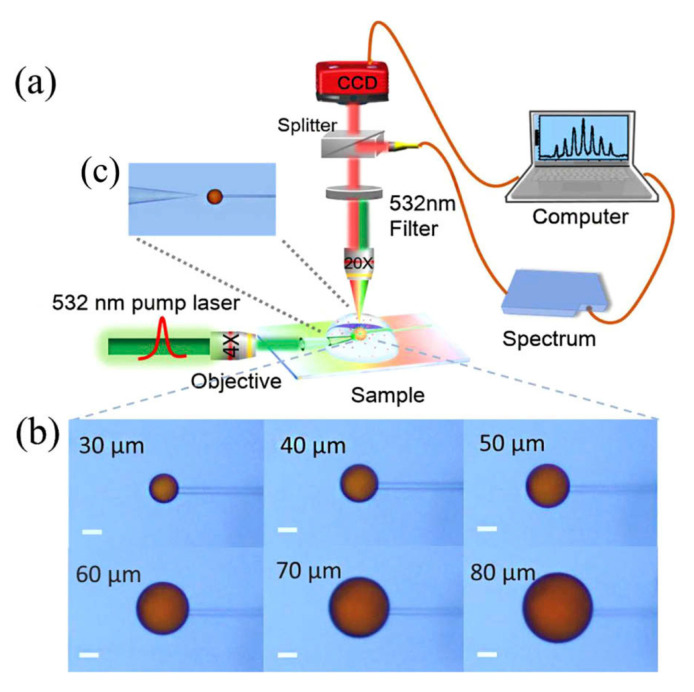
(**a**) Schematic diagram of the whispering gallery mode (WGM) experimental platform. (**b**) Micrographs of the stearic acid-doped 5CB microdroplets with different diameters. (**c**) Micrograph of microdroplet excited by a fiber tip positioned correctly within its vicinity. Reproduced from [36].

Currently, resonators are scarcely used in the detection of water pollutants. Figure 2 illustrates the work by Duan et al., in which liquid crystal microdroplets doped with stearic acid and LC 4-cyano-4′-pentylbiphenyl (5CB) were employed in WGM resonators for the detection of copper in drinking water [36].

#### 3.2.4. Gratings

Gratings are structures that are deposited on a specific surface in varying and periodic patterns. The presence of these structures results in a change in the refractive index [82]. The diffraction effect dominates if the index variation has a period greater than the wavelength of light within the grating. Otherwise, light propagation through the grating will display characteristics similar to those of the uniform medium, which will become increasingly pronounced as the period of the grating decreases [82]. The grating coupler can be a surface array, in which the light is coupled in the direction of the index variation if the index varies only in one direction. Alternatively, the array can be a subwavelength grating (SWG), which is a grating whose period is small enough to suppress diffraction effects [82]. Gratings are used for biosensing by measuring the diffraction efficiency of the analytes in a solution. Detection can also be carried out by measuring the angle-resolved diffraction efficiency. These approaches are used in diffraction grating sensors and in grating-coupled waveguide sensors, respectively [83]. If the refractive index varies only in one direction, light is coupled in the direction of the index variation.

**Figure 3:**
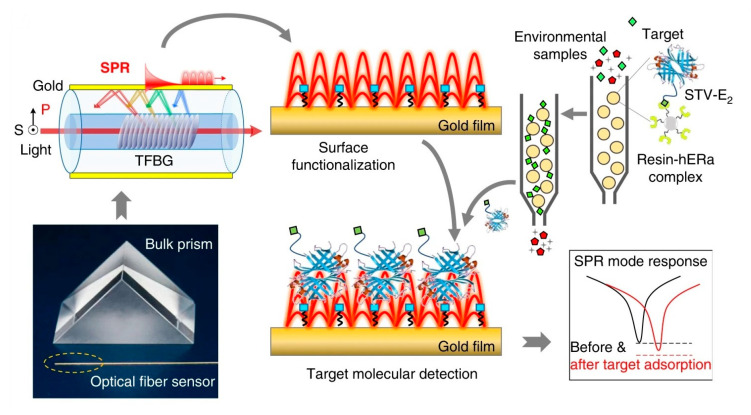
Plasmonic gold film-coated TFBG-based SPR biosensor for ultrasensitive estrogen (EE) detection. Reproduced from [34].

Optical gratings are easily coupled to other types of devices to improve their performance. For example, Liu et al. used a grating-coupled SPR to detect environmental estrogens (EE) in tap and bottled water [34]. As shown in Figure 3, SPR was carried out on a Tilted fiber Bragg gratings (TFBG), obtaining different additional resonance mechanisms, which were at wavelengths close to the near-IR. Resolution of the refractive index was improved by overlapping with the plasmon. In this biosensor, an estradiol-streptavidin conjugate was used as a recognition element.

#### 3.2.5. Fiber Optic

Among the advantages offered by fiber optics (FO) are immunity to electrostatic and electromagnetic interference, good biological compatibility, corrosion resistance and easy installation and operation [84,85]. Due to these characteristics, it is possible to find resonators, interferometers and SPRs developed with this material. However, by itself, fiber optics are also excellent materials, with optical properties that allow the detection of compounds. The simplest design of a fiber optics biosensor is to immobilize the biological elements in the coating; another option is to add subsequent layers to the coating that improve the performance of the biosensor.

#### 3.2.6. Fluorescence

Fluorescence is one of the most utilized transducers in biosensors [31]. Fluorescent materials can be used as sensing probes due to their ability to change their intrinsic fluorescence properties when interacting with other elements. The changes that occur during bio-recognition events are easily transformed into signals that are captured using different transducers [86]. Fluorescent biosensors can be constructed by measuring the intrinsic fluorescence of either the target molecule or the biological recognition element. Materials such as quantum dots (QD) can also be added to improve the fluorescence of the sensor [86].

Among the different fluorescence parameters that can be measured for biosensing are the following: (1) Intensity measures the spontaneous emission (fluorescence) after molecular excitation. The intensity of the light emitted at the analytical wavelength is directly related to the concentration of the fluorophore [87]. (2) Luminescence lifetime is the reciprocal of the rate constant of the emission decay that occurs when the luminophore is “instantaneously” excited by a flash of light. Lifetime measurements can be performed using a pulse of radiation with a width that is generally less than the luminophore’s decay period [88]. (3) Fluorescence quenching refers to any bi-molecular interaction that decreases the fluorescence intensity of the fluorophore molecule. One of the most problematic aspects of fluorometry is the high level of environment-dependent quenching. Nevertheless, if the fluorophore is the analyte, what was before considered a nuisance has now become a key use of fluorescence-based biosensors [87]. Finally, (4) Förster Resonance Energy Transfer (FRET) is a form of fluorescence quenching that happens when two distinct species—one (a donor) with a fluorescence spectrum that overlaps the excitation spectrum of the other (the acceptor)—are close enough to one other [52]. As a result, the radiation-excited donor can transfer energy non-radiatively to the acceptor, partially quenching the former’s fluorescence intensity, regardless of whether the acceptor is fluorescent or not. Thanks to this diversity of quantification methods, it is possible to increase the performance of biosensors in several ways.

#### 3.2.7. Comparison of Transducers Used in Optical Sensors for Monitoring Water Quality

Among the works for the detection of contaminants in water analyzed herein, biosensors based on interferometry stand out. These types of biosensors have allowed for the detection of pesticides, pharmaceuticals, and pathogenic microorganisms, where most achieve LOD below 1 µg/L.

A main advantage of SPR-based biosensors is their relatively simple configuration, i.e., they do not incorporate any other elements besides gold and the biological receptor. However, additional layers can be added to an SPR sensor to change the properties of the plasmon, allowing considerable signal and sensitivity improvement. One of the benefits of LSPR is that it allows us to experiment with different types, materials, and geometries of nanoparticles. An example of an LSPR biosensor for the detection of contaminants in water is the one developed by Kim and Lee in which gold nanostars are used in conjunction with a combination of aptamers and antibodies for the detection of an antibiotic [53]. In general, SPR and LSPR biosensors have low detection limits below 1 µg/L.

Resonators are not commonly used in the detection of water pollutants, as only two works with these types of biosensors were found, specifically for the detection of pesticides and heavy metals. Both works employed a whispering gallery mode configuration and reached LODs of 0.001 ug/L. In a similar manner, grating couplers are not as common in the development of water quality biosensors. However, there are two examples of their use for the detection of pathogens [50] and pesticides [6], and have also been shown to have low detection limits, being comparable to those mentioned above.

Some examples of fiber optic biosensors used in water quality are the tapered fiber optic designed by Arjmand et al. for pesticide detection [41] and the U-bent developed by Lamarca et al. for antibiotic detection [47]. Both have simple designs, which is an advantage for commercialization, and both have low LODs.

Finally, the use of fluorescence has been extended to all types of analytes, pesticides, pharmaceuticals, microorganisms, heavy metals, and organic compounds. In general, fluorescence biosensors have high LODs, with the lowest being ten to thousands of ug/L. To achieve LODs that compete with other biosensors, it has been necessary to replace traditional fluorometers with more efficient devices. Such is the case of the biosensors designed by Liu et al., in which FO and planar waveguides are used [89]. Another example is the biosensor based on Förster resonance energy transfer, which was also functionalized with graphene [52]. These devices were able to decrease the LOD down to 0.001 ug/L.

## 4. Detection of Selected Water Pollutants

### 4.1. Pesticides

Due to their frequent use in cultivation, pesticides reach surface and groundwater bodies during irrigation and precipitation. For example, Atrazine is one of the most ubiquitous pesticides, as its slow degradation makes it persistent, and its presence has been detected in water bodies worldwide [90]. Atrazine is also an endocrine-disrupting compound (EDC) that affects the sexual development of fishes, amphibians, reptiles, and mammals [91]. In addition to atrazine, organophosphate compounds have toxic effects at the neuronal level [92].

**Figure 4:**
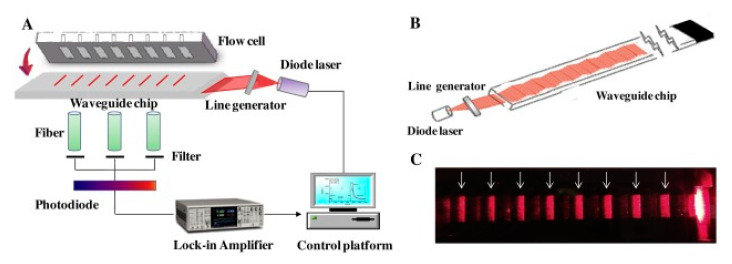
Schematic diagram of (**A**) a prototype array fluorescent biosensor PWAI with a removable multi-channel PDMS flow cell; (**B**) excitation light path inside the planar waveguide transducer; (**C**) photograph of waveguide chip with eight biosensing areas indicated by white arrows. Reproduced from [89].

Recent research for quantifying pesticides in water via optical biosensors employ different approaches. For example, a fluorescence biosensor with a planar waveguide-based array immunosensor (PWAI) was designed to be able to analyze multiple samples in a single measurement. This biosensor uses a planar waveguide that disperses light into different individual channels (see Figure 4), and detection is performed using fluorophore-labeled antibodies. One of the analytes tested was 2,4-dichlorophenoxyacetic acid (2,4-D), a commonly used herbicide, for which a limit of detection (LOD) of 7.53 μg/L was obtained [89].

Whole cells (i.e., encapsulated algae) have been used to measure chlorophyll fluorescence differences in the cells when exposed to pesticides. Tests were conducted with three different types of algae and three different pesticides, and the best result was a limit of detection of 10 μg/L [55]. Additionally, Scognamiglio et al. [90] presented an alternative whole-cell biosensor, where photosynthetic algae were immobilized on a paper base sensor to measured changes in fluorescence. This biosensor was used for the detection of atrazine, and an LOD of 80 ng/mL was obtained, proving it to be an efficient and sustainable biosensor thanks to the manufacturing materials.

A different approach is the use of grating couplers complemented by an immunoassay; this study was performed using atrazine as the target molecule resulting in an LOD of 0.05 μg/L [6]. Arjmand et al. [41] employed a tapered fiber optic enzyme biosensor for the detection of methyl-parathion, a highly toxic pesticide. With this approach, an LOD of 63.17 ng/L was obtained.

A research group in Greece developed an immunosensor with a simple arrangement to measure interference, and chlorpyrifos, thiabendazole and imazalil were simultaneously detected in water and wine samples. The results obtained were of LODs between 30–40 pg/mL [93]. Subsequently, the same methodology was used to detect atrazine and paraquat, with LODs of 0.04 ng/mL and 0.05 ng/mL, respectively [94].

A liquid crystal resonator has been developed for the detection of pesticides through the presence of enzymes and their inhibitors. This biosensor was tested with two analytes, and the limit of detection reached was 0.1 pg/mL for phenobucarb and 1 pg/mL for dimethoate [42]. More recently, an interferometer was developed for the detection of fenitrothion; this biosensor was based on bimodal waveguides and, using an immunoassay, was able to obtain an LOD of 0.29 ng/mL [46].

As observed in Table 2, most of the recent studies focused on pesticide detection in water have used fluorescence or interferometry, with the latter showing the best detection limits. Interferometers achieve high sensitivity and low detection limits, which makes them an analytical technique that can easily compete with chromatography. On the other hand, in the most recent work, a resonator was developed that was able to detect even lower concentrations. However, the advantage of the interferometer over the resonator is that its configuration is simpler.

### 4.2. Pharmaceuticals

Pharmaceutical compounds reach the environment through the incorrect disposal of their residues or through excretion. Like pesticides, these compounds are persistent and accumulate in the environment; some also have estrogenic effects in specific organisms and even alter the development of certain species of algae and microalgae [97]. In addition, the presence of antibiotics in water exacerbates the problem of increasing antibiotic-resistant bacteria [98].

SPR and LSPR are commonly employed for the detection of pharmaceuticals in water. For example, Tomasseti et al. [99] developed an immunoassay in a sandwich format for the quantification of ampicillin, obtaining an LOD of 0.3 g/L and showing it to be a biosensor with low sensitivity but good selectivity. One of the innovations in this kind of biosensors is the fabrication of the sensor chip, as was shown in Steinke et al. [100], where glass wafers with imprinted nanopillars were covered with gold and functionalized. This work was carried out for diclofenac detection, and an LOD of 1 μg/L was obtained. SPR can also be used in conjunction with other techniques to improve the sensitivity of the biosensor. An example of this is the biosensor designed by Altintas et al. [101], where molecularly printed polymers were deposited on the surface of the sensor chip. In this work, the detection of metoprolol in drinking water samples was performed, first in a simple way and then by adding gold nanoparticles to the samples so that the LOD improved from 78 μg/mL to 1.9 ng/mL.

Another SPR biosensor with a molecularly printed polymer was developed for the detection of ciprofloxacin, an antibiotic. The detection limit for this biosensor was estimated at 0.08 μg/L [102]. One of the most interesting advances is the biosensor developed by Shrivastav et al. [57], in which SPR and LSPR were combined with fiber optics. In this case, the biosensor was composed of an optical fiber with the core exposed in a section. A layer of silver was deposited onto the core, followed by silver nanoparticles; in this way, the different plasmons were enhanced, resulting in a highly sensitive biosensor. Tetracycline was used as target molecule, obtaining an LOD of 0.97 μg/L.

A similar work published that investigated the detection of tetracycline proposed a SPR/LSPR biosensor with an immunoassay in a sandwich format that integrated gold nanostars and a DNA aptamer. In this work, no limit of detection was established; however, it is reported that the biosensor was able reach attomolar concentrations [53]. A more recent study was conducted to detect the presence of ciprofloxacin in the effluent of water treatment plants. This work was carried out with an optical fiber immunosensor; the result obtained was an LOD of 3.3 × 10^−3^ ng/L [47].

The latest attempt to detect antibiotics by fluorescence combines a fiber optic sensor with molecularly imprinted composite hydrogel nanoparticle detector. The biosensor was used for the quantification of ciprofloxacin, for which a 6 μM LOD was obtained [58]. Diclofenac is another compound that has been detected using fluorescence; this biosensor was developed with yeast cells that fluoresce in its presence. The detection limit determined was 10 μM [54]. A different approach to the detection of antibiotics was developed by Weber et al. [103] using an interferometer for the quantification of penicillin, with 0.25 μg/mL being the minimum concentration tested.

**Figure 5:**
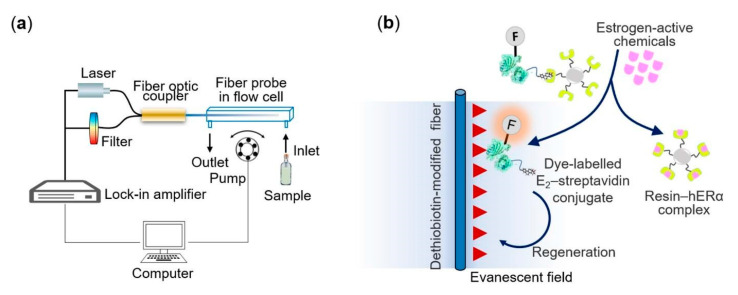
(**a**) Schematic of the optical-fiber biosensing platform. (**b**) Sensing scheme of the estrogen receptor-based biosensor using triple functional fluorescein-labelled E2–STV conjugates for estrogenic activities quantification. Reproduced from [33].

Detection of estrogens in water has received high interest because of their endocrine-disrupting effect on some marine species [33]. The hormone 17β-estradiol is one of the most widely used hormones for the development of biosensors. One of these studies was conducted by measuring the change in fluorescence intensity in water samples using a fiber optic biosensor that detected fluorescence by evanescent wave. The signal emitted was in response to an estrogen receptor (ER) binding with Cy5.5-labeled streptavadin, as observed in Figure 5. The result was an LOD of 1.5 μg/L, using 17β-estradiol as reference [33].

SPR has also been used for this same analyte, both in a classical Kretchmann configuration and in a format combining gratings and SPR mounted on an optical fiber. The latter performed an assay analyzing the interactions between 17β-estradiol and nuclear ER, obtaining an LOD of 0.0015 ng/mL [34], while the classical one used a competitive immunoassay format, achieving 1 pg/mL as the LOD [104].

Overall, SPR biosensors has been the most used technique for pharmaceutical detection (see Table 3). Fiber-optic biosensors also stand out, as they offer the lowest LOD using a simple configuration, so more research efforts in this area are pertinent.

### 4.3. Other Organic Compounds

This classification includes plastic derivatives, industrial supplies, fuels, detergents, or personal care products. Among them are phenolic and halogenated compounds. These attract special attention due to the harmful effects they have on aquatic organisms and even on humans [110].

**Figure 6:**
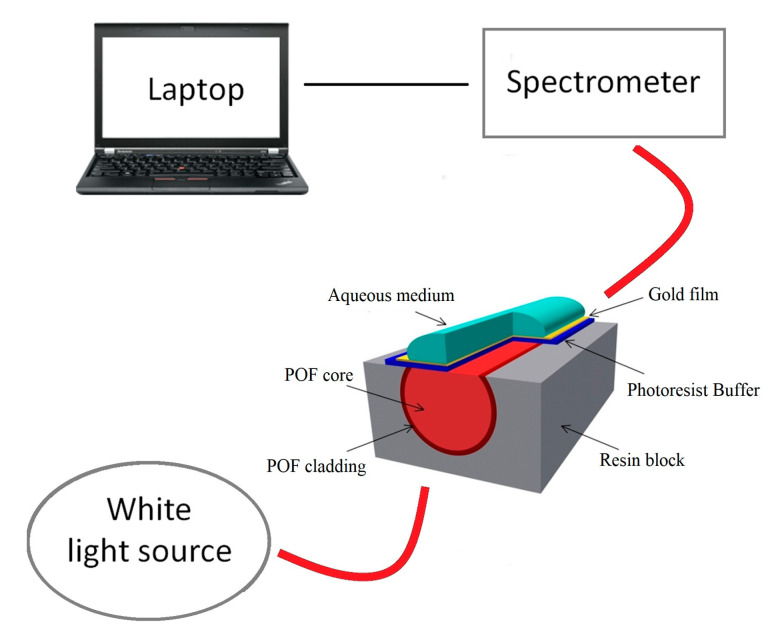
Schematic of the SPR-POF biosensor developed for the detection of naphthalene in water. Reproduced from [49].

Shahar et al. [44] proposed a biosensor based on an enzymatic membrane for the detection of organohalide, a halogenated compound, through an optical-fiber reflectometer. Under this scheme, an LOD of 0.908 mg/L was obtained. Furthermore, Cennamon et al. [49] proposed an SPR immunosensor for the detection of naphthalene. This immunosensor was designed using a plastic optical fiber (POF) with an exposed core, where a polymer layer and a gold layer were deposited (Figure 6). The advantages of this type of SPR-POF biosensor are ease of manufacture, installation, and use, as well as a larger fiber diameter. In this work, the presence of naphthalene in seawater was reported, with an LOD of 0.76 ng/mL.

Another compound that has been detected via SPR biosensors is bisphenol A (BPA). BPA is used in plastic manufacturing and can act as an endocrine disruptor. In this work, functionalized gold nanoparticles and an inhibition format were used; as a result, a detection limit of 5.2 pg/mL was obtained [48]. The previously mentioned PWAI fluorescent biosensor was also used for the detection of BPA; for this compound, an LOD of 0.03 μg/L was obtained [89]. Another way to use fluorescence as an analytical technique is through Förster Resonance Energy Transfer (FRET). This technique is based on the mechanism of energy transfer in a biological system. FRET was used in conjunction with graphene to develop a biosensor for the detection of BPA; this work resulted in a detection limit of 0.1 ng/mL [52]. Cheng et al. developed a biosensor that uses a smartphone to measure changes in the fluorescence of an immunoassay; the data are processed in an app, where the measurement can be tracked in real time. The immunoassay was performed with antibodies labeled with the Cy5.5 dye and was tested on water samples from a lake and tap water for the detection of BPA, achieving an LOD of 0.1 nM in terms of free Cy5.5 [18]. In another work, a simpler fluorescence array was used to test the detection of halogenated compounds. In this study, they tested for five different compounds, for which they obtained LODs in a range between 12.1 and 1.4 mg/L [43].

Table 4 includes the most recent studies published on the detection of organic compounds in water samples. Two works carried out with fluorescence can be seen that have the lowest detection levels. However, the configuration of these biosensors is not simple, and they also require the use of florescent markers. On the other hand, there are the SPR biosensors, which have a good performance, have simpler configurations, do not require markers and their detection limits are competent in relation to chromatography. Finally, there are the fiber-optic biosensors, which have a good detection limit, although they can be improved.

### 4.4. Microorganisms and Toxins

The presence of microorganisms has special interest in water quality monitoring since these are transmitters of diseases. Likewise, metabolites from some species of fungi contain harmful toxins.

Masdor et al. [114] used a simple SPR system for the detection of *Campylobacter jejuni* via a direct immunoassay, where an LOD of 8 × 10^6^ CFU/mL was obtained. Subsequently, an immunoassay in a sandwich format was performed, which improved the sensitivity of the biosensor, resulting in an LOD of 4 × 10^4^ CFU/mL. An alternative to the traditional SPR is SPR imaging (SPRi). It differs in that the detector is replaced by a CCD camera, which allows us to obtain a complete image of the sensing area. SPRi was employed by Foudeh et al. in the development of a biosensor for the detection of *L. pneumophila*. In this work, genetic material was used in conjunction with quantum dots. The biosensor was tested on water samples coming from a cooling tower, resulting in a 3 × 10^4^ CFU/mL LOD [115].

A more elaborate set-up was developed for the detection of *E. coli* O157:H7 in water and juice samples, which resulted in an LOD of 5 × 10^2^ CFU/mL [116]. The SPR fiber-optic biosensor had an optical fiber to which a mixture of silver nanoparticles with graphite was added, and then gold nanoparticles and a final gold film on which the biological element was deposited [116]. A different approach for the detection of *E. coli* is the biosensor designed by Sanati et al. [117], consisting of two resonator rings of different diameters arranged in a vernier structure, which improves the sensitivity of the measurement. This biosensor was tested with tap water samples by means of an immunoassay, for which an LOD of 3.33 × 10^−5^ RIU is reported.

**Figure 7:**
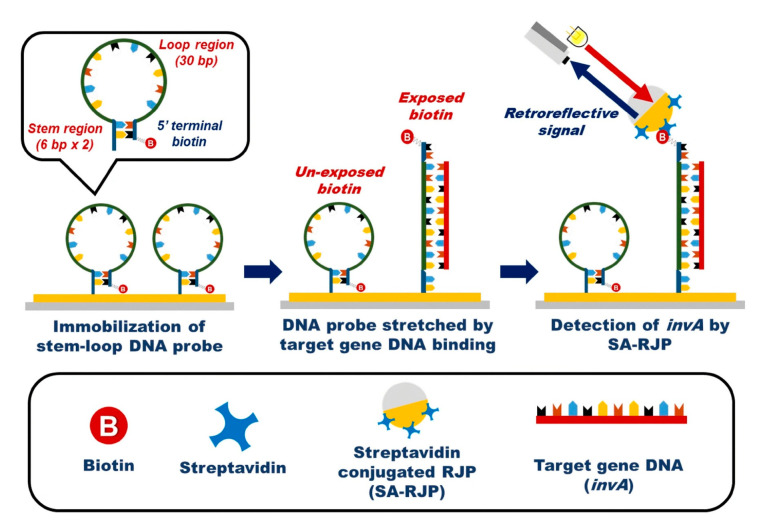
Schematic illustration of the *Salmonella* target gene DNA system detection using retroreflective Janus microparticles. Reproduced from [118].

In a different approach for *E. coli* detection, a porous silicon substrate was used in conjunction with lectin for the detection of *E. coli* and *Staphylococcus aureus*, obtaining an LOD of 103 cells/mL [15]. Another attempt to detect the presence of *S. aureus* in the environment led to the development of a long-period fiber grating immunosensor. This biosensor obtained an LOD of 244 CFU/mL [50]. Moreover, in an alternative technique to fluorescence, the retroreflector, which reflects light back to the source, has been used for bacterial detection. With this technique, the presence of *Salmonella typhimurium* was quantified using a stem-loop DNA modified with biotin, as shown in Figure 7. The biosensor was tested on synthetic samples and reached an LOD of 2.84 pM [118]. More recently, a monolithically integrated silicon interferometer was developed and used in conjunction with an immunoassay for the detection of *E. coli* and *S. typhimurium* in drinking water. This biosensor achieved an LOD of 40 CFU/mL for *S. typhimurium* and 110 CFU/mL for *E. coli* [119].

In the case of toxins, interferometers have been used in conjunction with an immunoassay for the quantification of ochratoxin A, a mycotoxin produced by more than one species of fungus. This biosensor reached an LOD of 0.01 ng/L [51]. Another interferometer for this purpose is the one made by Nabok et al. [120]. The sensor’s principal component is an optical waveguide made up of a thin silicon nitride layer inserted between two thicker silicon dioxide layers. The biosensor was tested on synthetic samples for the detection of zearalenone mycotoxin and reported an LOD of 0.01 ng/L. Microcystin-LR is a toxin produced by cyanobacteria and is one of the most toxic. Its detection was made in water samples with the PWAI biosensor. In this case, an LOD of 0.67 μg/L was obtained [89].

Table 5 summarizes recent publications on the detection of pathogenic microorganisms and toxins in water. Since the units in which the detection limits are reported are varied and their homogenization is somewhat complicated, it is difficult to make a correct comparison between them. However, the interferometer and the grating stand out because they seem to be the most sensitive and have low detection limits; therefore, it would be interesting to carry out more research using these techniques and exploit their potential.

### 4.5. Heavy Metals

Heavy metals such as copper, mercury, lead and cadmium are highly dangerous pollutants because they are not chemically or biologically degradable, which causes them to accumulate in soil, water, and air. Human exposure to heavy metals leads to serious diseases, from kidney failure [126] to cancer [36].

**Figure 8:**
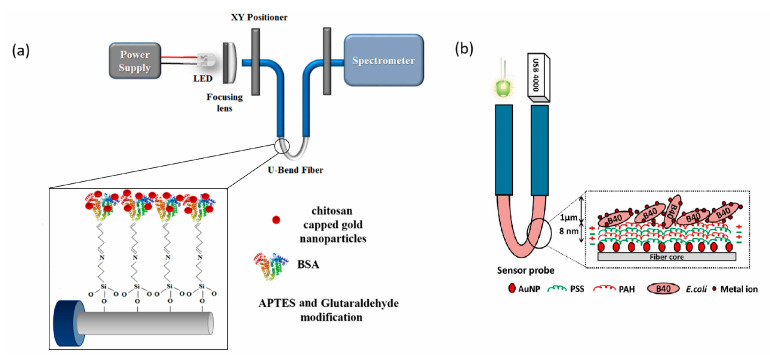
(**a**) Schematic of experimental set up and optical fiber modification with chitosan capped gold nanoparticles. Modified from [85] and (**b**) Schematic of the U-bend optical fiber biosensor for bacteria based heavy metal detection. Modified from [56].

In recent years, several types of optical biosensors have been developed for this purpose. An innovative and simple device was proposed by Tagad et al. [45]. In their work they developed an enzymatic biosensor using optical fiber to measure transmittance. This was used for the detection of Hg and resulted in an LOD of 2.5 μM. Years later, Sadani et al. proposed a U-bend fiber optic LSPR biosensor for the detection of Hg, which was functionalized with gold nanoparticles covered with chitosan, as shown in Figure 8a [85]. Meanwhile, Halkare et al. [56] used a similar U-bend fiber optic LSPR biosensor with immobilized *E. coli* cells (Figure 8b), which was able to detect Hg and Cd. Both biosensors were tested with tap water samples, and both achieved detection limits in the ppb range.

A different approach for detecting these compounds is the fluorescence biosensor. The first attempt to use this technique in the detection of heavy metals was by DNA attached to fiber optics. This study was conducted to quantify Hg and Pb, obtaining detection limits of 22 pM and 20 nM, respectively [126]. Several years later, fluorescence was employed to measure Hg via a cell-free expression system. The result was considerably superior, with a LOD of 1 ppb [127]. Similarly, Cu has been analyzed using a resonator, reaching an LOD of 40 pM [36].

Recently, the use of FPI for the detection of metals in water was reported. Such is the case of the biosensor developed by Bismuth et al. [128], in which they used porous silica functionalized with multiple layers of polyethylenimine for the detection of Cu. The biosensor was tested with irrigation, ground and tap water samples, showing an LOD of 53 ppb. Moreover, Kumar et al. [129] designed an FPI with DNAzyme, which contained a catalytic region and whose counterpart was coupled to silicon nanoparticles. This was used in ground, irrigation and tap water samples, reporting an LOD of 0.49 ppb.

According to Table 6, the lowest reported LOD corresponds to a WGM-type resonator made with liquid crystals. WGM has a great potential for the detection of compounds at low concentrations; however, few works have been developed in this area. Moreover, the fluorescence biosensors had a good response and low detection limits, so it can be considered as a simpler alternative.

## 5. Discussion

**Figure 9:**
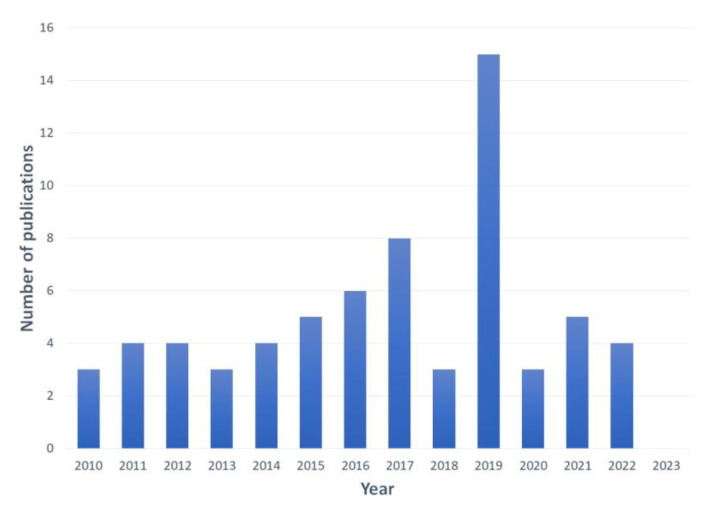
Yearly scientific production regarding biosensors for the detection of pollutants in water.

In this review, 67 papers on optical biosensors applied to the detection of various environmental water pollutants were compiled and considered for analysis. As observed in Figure 9, between the years 2010 and 2014, the number of publications was steady and started to consistently increase between 2015 and 2017. Most of the recent works were published in 2019, and this was followed by a marked decline, suggesting that due to the COVID-19 pandemic, the effort for biosensor development was even more concentrated in healthcare applications.

**Figure 10:**
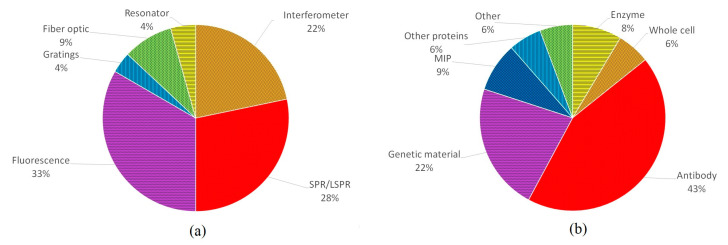
Percentage distribution of the works consulted according to (**a**) the type of biosensor and (**b**) the biological recognition element.

Figure 10 shows the distribution in terms of (a) the type of biosensor and (b) the biological recognition element. Regarding the type of biosensor, SPR and fluorescence biosensors represent 61% of the literature. As mentioned above, fluorescence is a preferred method because fluorescence transducers allow the measurement of different parameters, and the devices can have a wide variety of configurations. SPR devices can also be developed in various configurations, with the advantage of not using chemical markers (i.e., fluorescent labels). Both transducers can improve their sensitivity with the addition of other elements, e.g., multi-metal or other material layers, nanoparticles, quantum dots, etc. Due to the simplicity of setup and the detection limits they provide, these two transducers have been the most widely used so far.

Depending on their configuration, fluorescence-based biosensors can have detection limits varying from hundreds of ppb to less than one, and those that stand out will generally have more complex designs and operating principles. Such is the case for a fluorescence-based biosensor mounted on optical fiber or the use of FRET. However, these biosensors are usually preferred due to their relative simplicity compared to other optical biosensors.

SPR biosensors usually provide low detection limits, although this tends to be limited by the type of immunoassay. Even so, these limits can be further reduced by including other materials (e.g., metal nanoparticles, graphene, optical fiber) to improve device sensitivity. Moreover, SPR has become widespread since the optical arrangement and device has been thoroughly study and is relatively simple. Moreover, an SPR sensor is reusable, so multiple measurements can be performed.

Interferometers have also been shown to achieve low detection limits. Therefore, they account for 27% of the biosensors developed for the detection of contaminants in water. These types of biosensors can also be easily constructed and do not require markers. Meanwhile, fiber optics and grating couplers are often used in biosensor development in combination with other biosensing techniques, such as SPR and fluorescence, which may seem to be of little relevance but are key elements in biosensor design. During the literature search for this review, it was noticed that at the beginning of the decade, resonators were just emerging and being tested, so most of the articles published in those years do not offer a concrete application. However, resonators have been demonstrated to have high sensitivity and achieve low detection limits in the detection of small molecules in water, accounting for 4% of the publications included here. So, it is worthwhile to exploit their potential for environmental applications.

As for biological receptors, antibodies are the most common (43%), followed by genetic material (22%) and MIPs (9%). The use of antibodies is so widespread due to their compatibility with any type of transducer, their high specificity, and their sensitivity. Moreover, they have higher stability than enzymes and can be used for many target analytes, as opposed to DNA. In addition, their affinity for their antigens improves biosensor performance, which—combined with their specificity and the fact that these bonds can be dissociated, allowing the reuse of the sensor—make antibodies the best option in terms of receptors.

So far, the use of whole cells has been limited to fluorescence and SPR. However, with the use of bioreporter cells, their use could be expanded to other transducers [136]; however, these bioreporters have a great performance in fluorescence biosensing [137]. Another receptor that is of great interest at present are MIPs; although their use is relatively recent, it has been shown that these synthetic receptors have the potential to perform as well as natural ones, with the advantages of reducing production costs, being more stable and offering a more homogeneous sensor functionalization [138,139].

In general, sample preparation for biosensors was minimal, consisting of simple filtration if necessary; otherwise, the sample was read directly. This represents a great advantage compared to chromatographic methods, whose sample preparation includes several filtrations, separations, concentration, and in some cases, derivatization, making the pretreatment the most complex part of the analysis. Furthermore, biosensors proved to be competitive with chromatography since their detection limits are similar [56,85,93].

## 6. Key Trends and Future Perspective

Recent developments in optical sensors have shown considerable improvements in terms of sensitivity, such as SPCE sensors developed for the detection of pollutants such as tannic acid in environmental water samples [75] and perindopril erbumine in water and blood plasma samples [140]. Although these examples did not include a biological recognition element in their sensing platform, SPCE biosensors have been developed for clinical applications [141], proving that this technique could be an excellent alternative for enhancing the sensitivity of environmental biosensors. It is also important to highlight that nanomaterial advances such as soret and cryosoret that promote the generation of hotspots can improve the sensitivity of the biosensors that incorporate nanomaterials [142,143]. With proper biofunctionalization, these types of sensors can offer high-quality platforms for the environmental field. On the other hand, the detection of single molecules is a powerful technology that avoids bulk averaging and provides direct information on individual molecules [144]. Although single-molecule biosensors do not yet outperform ensemble-averaged approaches in terms of sensitivity, they show considerable improvement in affinity [81]. Single-molecule biosensors have been extensively studied in the healthcare field [145], making them a source of future research if these developments are extrapolated to environmental detection.

An advance in terms of portability and miniaturization is the use of the optical components of smartphones and their implementation in biosensing. Cell phones are one of the technological tools whose progress has been accelerated [146], and one of the features in which more has been invested is the capture of better quality photographs, since it is one of the elements that the user considers when choosing a product. Taking advantage of the high-resolution camera as well as other sensors present in smartphones for photo capture and data acquisition translates into cost reduction in the manufacturing of optical sensing devices [147]. For example, in 2017, McCracken et al. designed a sensor that was able to detect BPA in water samples using fluorescence [148]. This sensor used the camera flash as the excitation source and the camera’s complementary metal-oxide-semiconductor (CMOS) sensor as the detector. Once the images were obtained, they were processed in RGB format using ImageJ software. The result was a detection limit of 1004 μg L^−1^, which is not sufficient to compete with other techniques. However, this sensor represents a first approach to the accessible detection of contaminants in water with cell phones.

Since sensing the data of the aforementioned devices are complex due to variations in light or image aberrations, data processing tools such as machine learning (ML) are useful [149]. ML employs computer systems to replicate human learning and offers an algorithm with the capacity to identify and gain knowledge about the environment. Complicated biological systems are inherently compatible with ML algorithms since they can find hidden patterns [150]. In general, ML can approximate three sorts of problems: classification, regression, and clustering [151]. ML implementation can increase optical biosensor performance by simplifying the examination of raw biosensor output data to approximate a solution to various challenges. For example, (a) classification may be used for detection; (b) regression can be used to forecast and prevent harmful occurrences; and (c) clustering can be used to identify groupings of data that share features [151]; the optical biosensor signals can be analyzed in real time to develop meaningful output pathways in the algorithms, differentiating between good and bad images [14]. Leveraging these tools has facilitated the development of smartphone optical biosensors, which means we are one step closer to the miniaturization of these devices and their use for in situ monitoring. Taking the McCracken sensor as an example [148], it may be inferred that sensor performance could be enhanced by implementing ML for data processing.

Today it is feasible to develop smartphone-based optical biosensors that have low detection limits. An example of this is the biosensor developed by Guo et al. [152], in which they used a strip functionalized with streptavidin-biotinylated DNA probes modified with gold nanoparticles. Once the sample is run, an image is captured by a smartphone and the result is displayed by means of an application. This biosensor achieved an LOD of 0.5 μg L^−1^, being a remarkable improvement.

Considering all the above, there is still a wide range of research ahead. On the one hand there are the new proven techniques in sensors that can be coupled to biological receptors. Considering the advances in nanomaterials with the promotion of hotspots by means of sorets and cryosorets, this represents an improvement in SPR systems and any other system in which nanomaterials are to be implemented. This also includes the use of SPCE for biosensor fabrication and single-molecule detection. All these methodologies need to be explored in environmental applications.

On the other hand, it is also necessary to continue the research and development of portable, simple, and functional systems. Compared to traditional biosensors, smartphone-based biosensors are straightforward and simple to use. Such evaluations are accomplished at a cheaper cost and may thus be utilized for non-laboratory assessments. For water monitoring, this type of biosensor has been developed in two ways: detection through image capture from a chip or sensor cartridge where the measurement to be read is previously carried out, or with a small and portable array, which wirelessly connects to the smartphone and reads the signals. The first one is perhaps the most practical in terms of portability and commercialization. However, the second offers greater versatility in the type of array that can be fabricated, testing different transducers and receivers and with the possibility of integrating nanomaterials and microfluidics.

Although everything up to this point has been projected at the laboratory level, biosensors are a growing market. Previously, there have been biosensors for water quality assessment that have gone on sale, such as Optiqua™ [113], which is an interferometer that uses antibodies to detect contaminants. The use of biological elements is complicated, but technologies such as Optiqua™ are proof that it is possible to overcome this challenge. Additionally, the advancement in the development of MIPs is another possible solution that will improve the development and commercialization of biosensors in the future. With the integration of these new methodologies, the development of portable biosensors capable of detecting low molecular weight compounds at low concentrations is possible.

## 7. Concluding Remarks

Throughout this review, different types of optical biosensors and their applications in environmental detection have been described. The biosensors are diverse since each one is designed according to the needs and objectives of each application. However, SPR-based biosensors are amongst the most versatile, and their use extends to almost all kinds of pollutants found in water. Similarly, fluorescence is another technique that has been successful in the detection of contaminants in water. Between these two types of biosensors, the SPR-based biosensors have an advantage over fluorescence since, in general, they can reach lower detection limits.

Other types of devices, such as interferometers and resonators, are less commonly used but have been proven to have a high sensitivity and to have low detection limits. The potential shown by these types of biosensors makes it worthwhile to further develop them.

Similarly, each type of biological recognition elements has its advantages and disadvantages. Antibodies are the most versatile and widely used receptors because of their various advantages; however, it is necessary to continue developing research on this subject to make antibodies more affordable. Enzymes are very sensitive to changes in their environment, so this instability limits their potential, while DNA is limited in terms of the variety of analytes it can quantify.

Some of the challenges faced by optical biosensors for their commercialization is the miniaturization of the devices because their optical components are generally delicate and even more expensive than those used in electrochemistry. However, with the increasing development of technology, it is possible to develop biosensors that take advantage of the optical elements of smartphones to develop biosensors. Another challenge is to guarantee the stability of the biological element and to achieve the reusability of the sensors. Continuing to innovate and develop research in biosensors and related areas is of the utmost importance to overcome these barriers and achieve their widespread application for environmental analysis.

## Figures and Tables

**Table 1:** Comparison between different biological recognition elements.

Biological Element	Affinity	Specificity	Sensibility (LOD, ng L^−1^)	Stability	Versatility	Cost
Enzyme	High	Medium/high	12,000–1 × 10^−4^	Medium/low	High	Low
Antibody	High	High	250–0.07 × 10^−6^	High	High	High
DNA/RNA	Very High	Very high	4.14–4.4 × 10^−3^	High	Low	Low
Cell	Medium	Medium	2900–0.5	High	Medium	Low
MIP	High	High	1900–0.08	High	High	Low

**Table 2:** Published work on detection of pesticides in water.

Target Analyte	Type of Biosensor	Limit of Detection (ppb)	Sample	Bio	Reference
2,4-D	Fluorescence	7.53	Drinking water	Antibody	[89]
2,4-D	Fluorescence	2.17	Deionized water	Antibody	[95]
Diuron	Fluorescence	10	Deionized water	Cell	[55]
Isoproturon		10			
Atrazine		10			
Atrazine	Fluorescence	80	Tap water	Cell	[90]
Atrazine	Fluorescence	0.77	Lake water	MIP	[96]
Atrazine	Grating couplers	0.05	Tap and river water	Antibody	[6]
Methyl-parathion	Fiber optic	0.063	Deionized water	Enzyme	[41]
Chlorpyrifos	Interferometer	0.03	Spiked bottled water	Antibody	[93]
Thiabendazole		0.04			
Imazalil		0.03			
Atrazine	Interferometer	0.04	Deionized water	Antibody	[94]
Paraquat		0.05			
Fenitrothion	Interferometer	0.29	Tap water	Antibody	[46]
Phenobucarb	Resonator	1 × 10^−4^	River water	Enzyme	[42]
Dimethoate		1 × 10^−3^			

**Table 3:** Published work on detection of pharmaceutical compounds in water.

Target Analyte	Type of Biosensor	Limit of Detection (ppb)	Sample	Bio	Reference
Ampicilin	SPR	0.3 × 10^6^	Deionized water	Antibody	[99]
Tetracycline	SPR/LSPR	0.97	Deionized water	MIP	[57]
Tetracycline	SPR/LSPR	<ppb	River water	Aptamer and antibody	[53]
Metoprolol	SPR	1.9	Drinking water	MIP	[101]
Ciprofloxacin	SPR	0.08	Deionized water	MIP	[102]
Diclofenac	SPR	1	Deionized water	Antibody	[100]
17β-estradiol	SPR	0.001	Deionized water	Antibody	[104]
17β-estradiol	SPR/Grating	0.0015	Spiked tap and pond water	ER hERα	[34]
17β-estradiol	SPR	6.8 × 10^−5^	Wastewater	MIP	[105]
17β-estradiol	Fluorescence	0.14	Wastewater	DNA	[106]
Sulfadimine	Fluorescence	0.06	Wastewater, lake and bottled water	Antibody	[107]
Diclofenac	Fluorescence	2900	Wastewater	Cell	[54]
Ciprofloxacin	Fluorescence	1900	River water	MIP	[58]
Estrogen	Fluorescence	1.05	Wastewater	Estrogen receptors ERα and Erβ	[33]
Ciprofloxacin	Fiber optic	3.3 × 10^−6^	Wastewater	Antibody	[47]
Penicillin	Interferometer	250	Deionized water	Antibody	[103]
Amoxicillin	Interferometer	>1	Wastewater, lake and drinking water	Antibody	[108]
Ibuprofen	Interferometer	1000	Deionized water	Chitosan	[109]

**Table 4:** Published work on detection of organic compounds in water.

Target Analyte	Type of Biosensor	Limit of Detection (ppb)	Sample	Bio	Reference
Dichloroethane	Fiber optic	1000	River, tap and bottled water	Enzyme	[44]
Naphthalene	SPR	0.76	Sea water	Antibody	[49]
Bisphenol A	SPR	5.2 × 10^−3^	Deionized water	Antibody	[48]
Bisphenol A	SPR	0.14	Wastewater	Antibody	[111]
Bisphenol A	SPR	0.04	Drinking water	Antibody	[112]
1,2-dibromoethane	Fluorescence	2400	River water	Enzyme	[43]
1,2,3-trichloropropane		1400			
1,2-di-chloroethane		2700			
3-chloro-2-(chloromethyl)-1-propene		1400			
γ-hexa-chlorocyclohexane		12,100			
Bisphenol A	Fluorescence	0.03	Drinking water	Antibody	[89]
Bisphenol A	Fluorescence	0.001	River, tap and bottled water	DNA	[52]
Bisphenol A	Fluorescence	0.076	Lake and tap water	Antibody	[18]
Bisphenol A	Fluorescence	0.025	Deionized water	Antibody	[95]
Bisphenol A	Interferometer	0.5	Treated water	Antibody	[113]

**Table 5:** Published work on detection of microorganisms and toxins in water.

Target Analyte	Type of Biosensor	Limit of Detection	Sample	Bio	Reference
Microorganisms					
*L. pneumophila*	SPRi	3 × 10^4^ CFU/mL	Spiked water from a cooling tower	DNA	[115]
*C. jejuni*	SPR	4 × 10^4^ CFU/mL	Deionized water	Antibody	[114]
*E. coli* O157:H7	SPR	5 × 10^2^ CFU/mL	Spiked tap water	Peptide	[116]
*E. coli*	Interferometer	10^3^ cells/mL	Deionized water	Lectins of Concanavalin A	[35]
*S. aureus*		10^3^ cells/mL			
*E. coli*	Interferometer	110 CFU/mL	Drinking water	Antibody	[119]
*S. typhimurium*		40 CFU/mL			
*E. coli*	Resonator	3.33 × 10^−5^ RIU	Drinking water	Antibody	[117]
*E. coli*	Fluorescence	10 CFU/mL	Wastewater	DNA	[121]
*S. aureus*	Grating	244 CFU/mL	Deionized water	Antibody	[50]
*S. typhimurium*	Retroreflector	2.84 pM	Deionized water	DNA	[118]
Toxins					
Microcystin-LR	Fluorescence	0.67 ppb	Drinking water	Antibody	[89]
Microcystin-LR	Fluorescence	0.03 ppb	Deionized water	Antibody	[95]
Microcystin-LR	Fluorescence	0.016 ppb	Fresh water	Antibody	[122]
Microcystin-LR	Fluorescence	0.14 ppb	Drinking water	DNA and antibody	[123]
Microcystin-LR	Fluorescence	0.09 ppb	Lake water	Antibody	[124]
Microcystin-LR	Fluorescence	0.5 ppb	Lake water	DNA	[125]
Microcystin-RR		0.3 ppb			
Ochratoxin A	Interferometer	1 × 10^−3^ ppb	Deionized water	Antibody	[51]
Zearalenone	Interferometer	0.01 ppb	Deionized water	Antibody	[120]

**Table 6:** Published work on detection of heavy metals in water.

Target Analyte	Type of Biosensor	Limit of Detection (ppb)	Sample	Bio	Reference
Hg	Optical fiber	5001	Deionized water	Enzyme	[45]
Hg	LSPR	0.1	Spiked seawater, wastewater and tap water	BSA and Chitosan	[85]
Hg	LSPR	0.5	Tap water	Cell	[56]
Cd		0.5			
Hg	SPR	0.01	Tap water	DNA	[130]
Hg	SPR	0.2	Tap and pond water	DNA	[131]
Hg	Optical fiber	4.4 × 10^−3^	Drinking water	DNA	[126]
Pb		4.14			
Pb	Optical fiber	0.21	Wastewater	DNA	[132]
Hg	Optical fiber	0.58	Wastewater, tap and bottled water	DNA	[133]
Hg	Fluorescence	1	Deionized water	DNA	[127]
Hg	Fluorescence	0.24	Surface water	DNA	[134]
Cu	Resonator	2.5 × 10^−3^	Drinking water	Stearic acid	[36]
Cu	Interferometer	0.53	Ground water, irrigation water and tap water	Polyethylenimine	[128]
Cu	Interferometer	104	Tap, irrigation and drain water	Enzyme	[135]
Ag		56			
Pb		125			
Pb	Interferometer	0.49	Ground water, irrigation water and tap water	DNA	[129]

## Data Availability

No new data were created or analyzed in this study. Data sharing is not applicable to this article.
